# Dietary Pectin Increases Intestinal Crypt Stem Cell Survival following Radiation Injury

**DOI:** 10.1371/journal.pone.0135561

**Published:** 2015-08-13

**Authors:** Sripathi M. Sureban, Randal May, Dongfeng Qu, Parthasarathy Chandrakesan, Nathaniel Weygant, Naushad Ali, Stan A. Lightfoot, Kai Ding, Shahid Umar, Michael J. Schlosser, Courtney W. Houchen

**Affiliations:** 1 Department of Medicine, The University of Oklahoma Health Sciences Center, Oklahoma City, Oklahoma, United States of America; 2 Department of Pathology, The University of Oklahoma Health Sciences Center, Oklahoma City, Oklahoma, United States of America; 3 Department of Biostatistics and Epidemiology, College of Public Health, The University of Oklahoma Health Sciences Center, Oklahoma City, Oklahoma, United States of America; 4 Department of Veterans Affairs Medical Center, Oklahoma City, Oklahoma, United States of America; 5 The Peggy and Charles Stephenson Cancer Center, Oklahoma City, Oklahoma, United States of America; 6 Department of Molecular & Integrative Physiology, The University of Kansas, Kansas City, Kansas, United States of America; 7 COARE Biotechnology Inc., Oklahoma City, Oklahoma, United States of America; University of Kentucky, UNITED STATES

## Abstract

Gastrointestinal (GI) mucosal damage is a devastating adverse effect of radiation therapy. We have recently reported that expression of Dclk1, a Tuft cell and tumor stem cell (TSC) marker, 24h after high dose total-body gamma-IR (TBI) can be used as a surrogate marker for crypt survival. Dietary pectin has been demonstrated to possess chemopreventive properties, whereas its radioprotective property has not been studied. The aim of this study was to determine the effects of dietary pectin on ionizing radiation (IR)-induced intestinal stem cell (ISC) deletion, crypt and overall survival following lethal TBI. C57BL/6 mice received a 6% pectin diet and 0.5% pectin drinking water (pre-IR mice received pectin one week before TBI until death; post-IR mice received pectin after TBI until death). Animals were exposed to TBI (14 Gy) and euthanized at 24 and 84h post-IR to assess ISC deletion and crypt survival respectively. Animals were also subjected to overall survival studies following TBI. In pre-IR treatment group, we observed a three-fold increase in ISC/crypt survival, a two-fold increase in Dclk1+ stem cells, increased overall survival (median 10d vs. 7d), and increased expression of Dclk1, Msi1, Lgr5, Bmi1, and Notch1 (in small intestine) post-TBI in pectin treated mice compared to controls. We also observed increased survival of mice treated with pectin (post-IR) compared to controls. Dietary pectin is a radioprotective agent; prevents IR-induced deletion of potential reserve ISCs; facilitates crypt regeneration; and ultimately promotes overall survival. Given the anti-cancer activity of pectin, our data support a potential role for dietary pectin as an agent that can be administered to patients receiving radiation therapy to protect against radiation-induces mucositis.

## Introduction

Radiotherapy is one of the three major treatment modalities used to eradicate malignant solid tumors. When ionizing radiation (IR) is used to treat abdominal and pelvic tumors, severe side effects (largely due to gut mucosal damage) are common. The resultant mucosal ulceration (mucositis), pain, and related sequel are a cause of significant morbidity [1]. Gastrointestinal mucositis results in symptoms, which include: nausea and vomiting; pain; diarrhea; constipation; increased frequency and urgency of defecation; and rectal bleeding [2, 3]. It affects between 40% and 100% of patients undergoing treatment for their cancer, causing distress, debilitation, and occasionally death [1, 4, 5]. Massive intestinal crypt loss results in an extensive breakdown of the gut mucosal barrier. Because IR also impairs the bone marrow-derived immune response, susceptibility to overwhelming infection causes significant morbidity and mortality. Prevention or treatment of radiotherapy-induced adverse effects would improve quality of life for patients and perhaps increase cancer curability by allowing for more intense therapies [6].

The adult intestinal epithelium is continuously and rapidly replaced by cell replication within the crypts of Lieberkühn, and subsequent migration of their progeny takes place onto the villus epithelium in the small intestine, or onto the surface epithelium in the colon [7, 8]. Doublecortin-like kinase 1 (Dclk1), is a microtubule associated protein kinase, that is predominantly expressed in quiescent cell populations at the +4 location of the intestinal crypt, and occasionally in crypt based columnar cells [9, 10]. Dclk1 is also expressed on villus epithelial cells and has recently been shown to mark Tuft/Brush cells [11, 12]. While their precise role is uncertain, recent publications have implicated Tuft/Brush cells in chemosensing through taste transduction-related proteins [11, 13]. Crypt Dclk1+ cells did not undergo apoptosis in the intestine at 6 h following 6 Gy total body IR (TBI) based on histological observation and terminal deoxynucleotidyl transferase dUTP nick end labeling (TUNEL) assay. It was further demonstrated that at 24h post 6 Gy IR, Dclk1+ cells underwent apoptosis and mitosis within the intestinal crypt region [9]. These data suggest that the 24h time point may be an ideal time point to assess the effects of IR on Tuft/brush cell deletion. Furthermore, although surviving Dclk1+ cells underwent mitosis 24h, 7 and 10 days post-IR, the cells retained BrdUrd labeling at the +4 position in the intestinal crypt, indicating that they underwent proliferation at least once following IR-induced injury [10]. These data suggest that Dclk1+ cells may act as rescue or reserve stem cells upon high-dose IR. Recently, we have demonstrated that mice with intestinal and colonic specific knockout of Dclk1 had an impaired regenerative response following high-dose TBI [14]. Dclk1 knockout mice died early compared to wild type littermates [14].

Agents given before the radiation treatments are termed radioprotectors, where as agents given after treatment are termed radiation mitigators [15]. Pectin is a highly-complex branched polysaccharide fiber, rich in galactoside residues and present in all plant cell walls [16]. A reduction in the growth of colon tumors implanted in mice after oral administration of pectin has been demonstrated [17]. However, the role of pectin as a radioprotector and its effect on stem cell survival has not been studied in detail.

Here, we report that dietary citrus pectin is a radioprotective agent, prevents IR-induced deletion of ISCs, facilitates crypt regeneration, and promotes overall animal survival. Furthermore, we observed increased expression of ISC-related genes Dclk1, Notch1, Lgr5, Bmi1, and Msi1 in the small intestine of mice treated with pectin compared to control animals, indicating their potential involvement role in the ISC niche response following radiation injury.

## Materials and Methods

### Experimental animals

Six to eight-week-old female C57BL/6 mice (The Jackson Laboratory, Bar Harbor, ME) were used in the experiments. Mice were housed under controlled conditions, including a 12-h light-dark cycle, with ad libitum access to food and water. This study was carried out in strict accordance with the recommendations in the Guide for the Care and Use of Laboratory Animals of the National Institutes of Health. The protocol was approved by the Committee on the Ethics of Animal Experiments—Institutional Animal Care and Use Committee (IACUC), the University of Oklahoma Health Sciences Center (protocol # 12-104-H). Animals were caged together in a Hepa filter topped cages to prevent infection, easy access to water and food, and all efforts were made to minimize suffering.

### Pectin treatment

For the ‘pre-IR’ experiments, adult C57BL/6 mice were placed on a 6% citrus pectin diet (TD.97202) [18] (Harlan Laboratories Inc., USA) with additional 0.5% pectin (Sigma-Aldrich, St. Louis, MO) in their drinking water for one week prior to IR. For ‘post-IR’ experiments, mice were placed on pectin diet and pectin water immediately after the IR treatments. Control animals received control diet (control diet had similar ingredients to that of pectin diet, but lacking pectin) (TD.97201) (Harlan Laboratories Inc., USA) and normal drinking water. All the animals in the pectin groups (pre-IR and post-IR) received pectin diet and pectin water until euthanasia or death.

### Gamma Radiation

**Fig 1:**
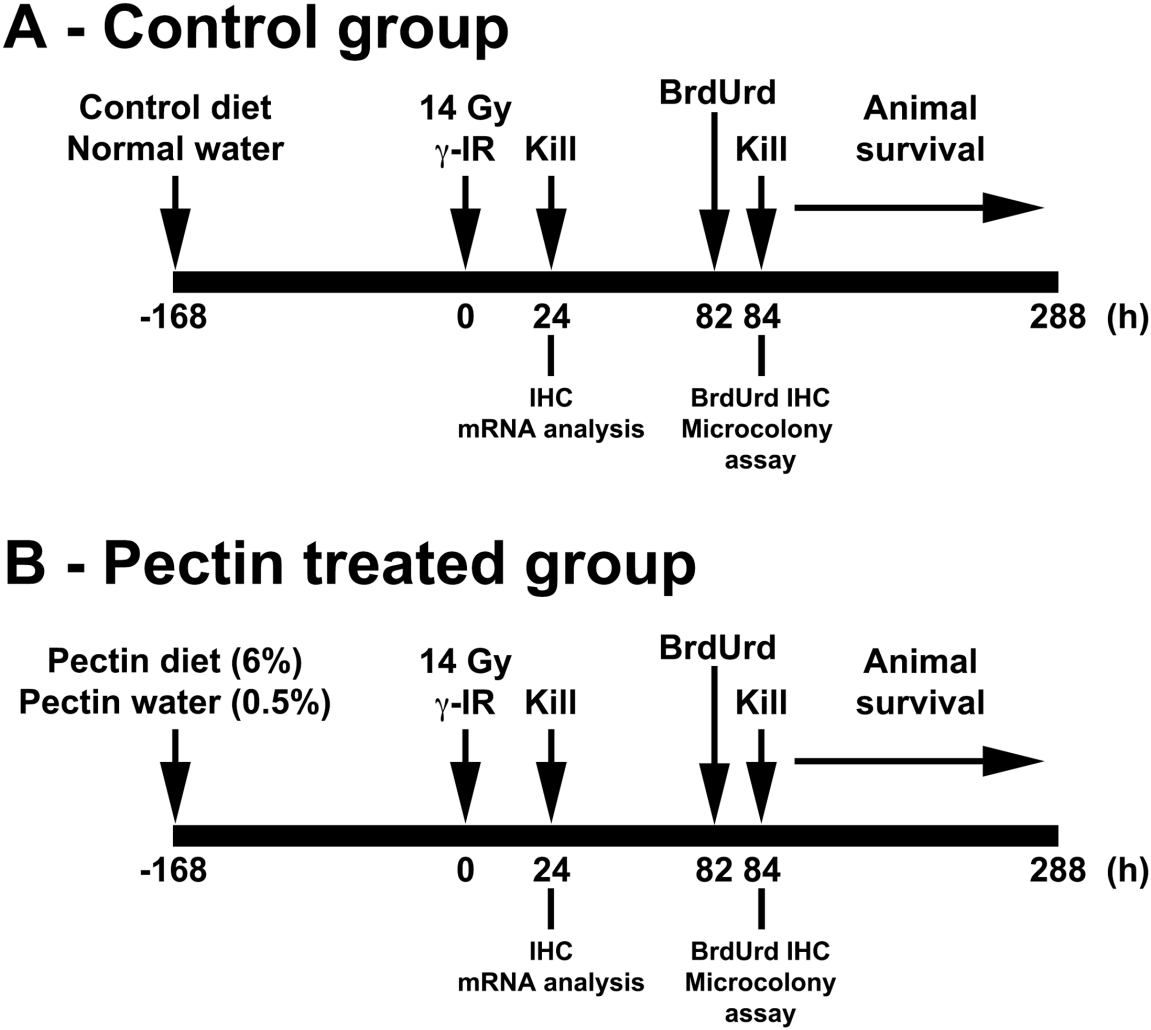
Schematic presentation of experimental design. C57BL/6 mice in the control group (A) or the pectin treated group (B) were fed with control or pectin diet 168h prior to 14 Gy TBI (n = 3 in each group). One set of mice were euthanized at 24h post-IR for IHC and mRNA analysis. A second set were given BrdUrd at 82h post-IR and then euthanized at 84h for the microcolony assay. The final set of mice was subjected to overall animal survival studies up to 288h post-IR—times of death were noted.

Mice were exposed to TBI 14 Gy gamma ionizing radiation (IR) with air pumped into the chamber during exposure. A Gammacell 40 ^137^Cs gamma irradiator was used with a dose rate of 0.8 Gy IR per minute. Dosimetry measurements were performed using Fricke Dosimetry systems. Measured absorbance dose was: Central Dose Rate (0.790 Gy/min ± 2.9%); 3 o’clock position (0.804 Gy/min ± 3.8%); and 7 o’clock position (0.808 Gy/min ± 2.7%). The experiments were performed within 6 months of the dosimetry analysis. All IR treatments were begun in the morning. Animals in pectin group continued to receive pectin post-IR. Two hours before euthanasia, each mouse was intraperitoneally injected with 5-bromo-2’-deoxyuridine (BrdUrd, Sigma Aldrich, St. Louis, MO, 200 μl of 5 mg/ml BrdUrd solution in PBS). The experimental design is schematically presented in Fig 1 [9, 10].

### Immunohistochemistry

Heat-induced epitope retrieval was performed on 4-μm formalin-fixed, paraffin-embedded sections utilizing a pressurized Decloaking Chamber (Biocare Medical LLC, Concord, CA) in citrate buffer (pH 6.0) at 99°C for 18 min. Slides were incubated in 3% hydrogen peroxide at room temperature for 10 minutes. After incubation with primary antibody (anti-Dclk1 c-terminal ab31704, 1:8000 dilution or anti-BrdUrd ab6326, 1:300 dilution [Abcam, Cambridge, MA]) overnight at 4°C, slides were incubated in Promark peroxidase-conjugated polymer detection system (Biocare Medical LLC) for 30 min at room temperature. After washing, slides were devolved with Diaminobenzidine (Sigma—Aldrich) [9, 10]. Slides were examined on the Nikon Eclipse Ti motorized microscope paired with the DS-Fi2 color digital cameras utilizing DIC enhanced PlanApo objectives operated by the NIS-Elements Microscope Imaging Software platform (Nikon Instruments, Melville, NY).

### Microcolony assay

Following the IR treatment, animals were euthanized at 3.5d (84h). The whole intestine was fixed and distal jejunum was subjected to BrdUrd immunostaining. The number and width of surviving crypts in the intestine were measured. A surviving crypt was defined as containing five or more adjacent, BrdUrd-positive nuclei [10]. The circumference of a transverse section of intestine was used as a unit of length, and the number of crypts in each circumference was recorded. Ten circumferences per mouse (0.5 to 1 cm apart) and three mice per experimental group were assessed [10]. All the 30 cross-sections were averaged together and represented.

### Animal survival study

Following the IR treatment mice in the pre-IR pectin and post-IR pectin groups were kept on pectin diet and pectin water and mice in control group were kept on normal diet and water and monitored for survival (n = 6 in each group). An animal was considered to be in distress if it experiences any one of the following: loss of 25% or more body weight, unthriftness (inability to walk, run, eat or drink properly) due to injury or becomes moribund or lethargic, were euthanized immediately. Those mice displaying bloody stools, wounds, or any other symptoms that affect posture or appear to be uncomfortable was euthanized immediately. Death is not the endpoint of the study and no surgeries were performed.

For euthanasia mice were given CO_2_ from gas compressed in cylinders. Death was confirmed by physical examination, ensured by an adjunctive physical method such as thoracotomy or cervical dislocation.

To minimize the sufferings, all the animals had easy access to food and water, were monitored every 6–8 hours, and weighted everyday. Overall survival was defined as the time interval between the completion of IR and the final euthanasia.

### Real-time polymerase chain reaction (RT-PCR) analyses

Total RNA isolated from mouse intestines (24h post-IR) (n = 3 per group and one sample per mouse) was subjected to reverse transcription with Superscript II RNase H—Reverse Transcriptase and random hexanucleotide primers. The complimentary DNA (cDNA) was subsequently used to perform real-time (RT) reverse transcriptase-PCR by SYBR chemistry using gene specific primers and JumpStart Taq DNA polymerase (Sigma-Aldrich, St. Louis, MO). The data was normalized with **β**-actin. The changes in mRNA were expressed as fold change relative to control with ± SEM value (* *p* < 0.01) [19–22]

The following primers were used:

**β**-actin: forward: 5'-GGTGATCCACATCTGCTGGAA-3',
  reverse: 5'-ATCATTGCTCCTCCTCAGGG-3';
Dclk1: forward: 5'- CAGCAACCAGGAATGTATTGGA -3',
  reverse: 5'- CTCAACTCGGAATCGGAAGACT-3';
Msi1: forward: 5’-CAGTTTCGGACCTATCTCTGAGGT-3’
  reverse: 5’-AAGGTGATGAAACCAAAACCCCT-3’
Notch1: forward: 5'-CGGGTCCACCAGTTTGAATG-3',
  reverse: 5'-GTTGTATTGGTTCGGCACCAT-3'.
Lgr5: forward: 5’-CAGTTTCGGACCTATCTCTGAGGT-3’
  reverse: 5’-AAGGTGATGAAACCAAAACCCCT-3’
Bmi1: forward: 5'-CGGGTCCACCAGTTTGAATG-3',
  reverse: 5'-GTTGTATTGGTTCGGCACCAT-3'.

### Statistical analysis

Real-time PCR Data was analyzed using the Student’s t test for comparison of mean values between groups. Results were reported as average ± SEM.

Since survival time was observed in every mouse (i.e., no censoring), side-by-side box plots were generated to graphically present the data. Median survival time and its 95% confidence interval (CI) for mice in Pectin and control groups were reported. These survival data were also summarized using the Kaplan-Meier curves and compared based on the log-rank test. The SAS software (version 9.3, Cary NC) was used for analyzing the survival data.

A two-sided p-value of < 0.05 defines statistical significance.

## Results

### Pectin increases crypt survival following IR injury

**Fig 2:**
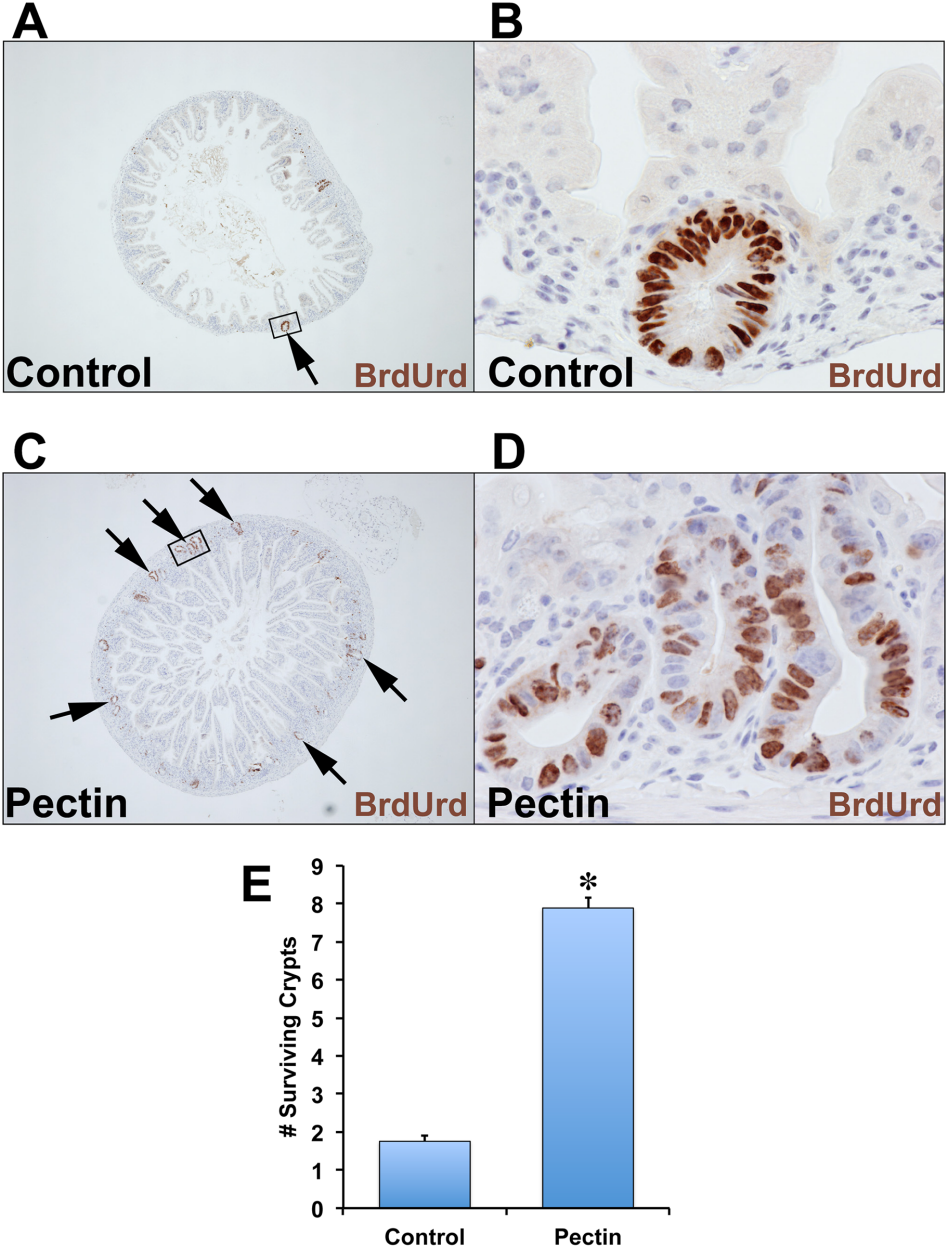
Treatment with pectin increased the number of surviving crypts 84h post-IR. C57BL/6 mice in the control group (A, B) or the pectin-treated group (C, D) were subjected to TBI (n = 3 in each group). 82 h post-IR, these mice were injected with BrdUrd, then euthanized at 84h post-IR. The small intestines were isolated, fixed, and processed. The presence of five to six BrdUrd positive cells grouped together indicated surviving crypts. Panel B is the high-powered magnified images of the panel A and panel D is the high-powered image of panel C. The average number of surviving crypts per cross section was counted for total of 30 cross sections and is presented in panel C. The values in the bar graph are given as average ± SEM and * denotes statistically-significant differences (*p<0.01) compared to control.

To investigate the effects of dietary pectin on radiation-induced ISC/crypt survival, we fed C57BL/6 mice with pectin diet for one week before subjecting them to 14 Gy TBI (n = 3). A schematic representation of the experimental design is provided in Fig 1. We observed an average of 1.48 surviving crypts per cross section in mice treated with IR alone (Fig 2A, 2B and 2E). In the pectin-pretreated animals, we observed a 4.5-fold increase in surviving crypts (7.88 crypts) per cross section (Fig 2B, 2D and 2E). Whereas in the pectin-posttreatment experiment, we did not observe any differences in the number of surviving crypts between the pectin-posttreated and control animals (data not shown).

### Pectin treatment increases animal survival following IR injury

#### Pre-IR pectin treatment

**Fig 3:**
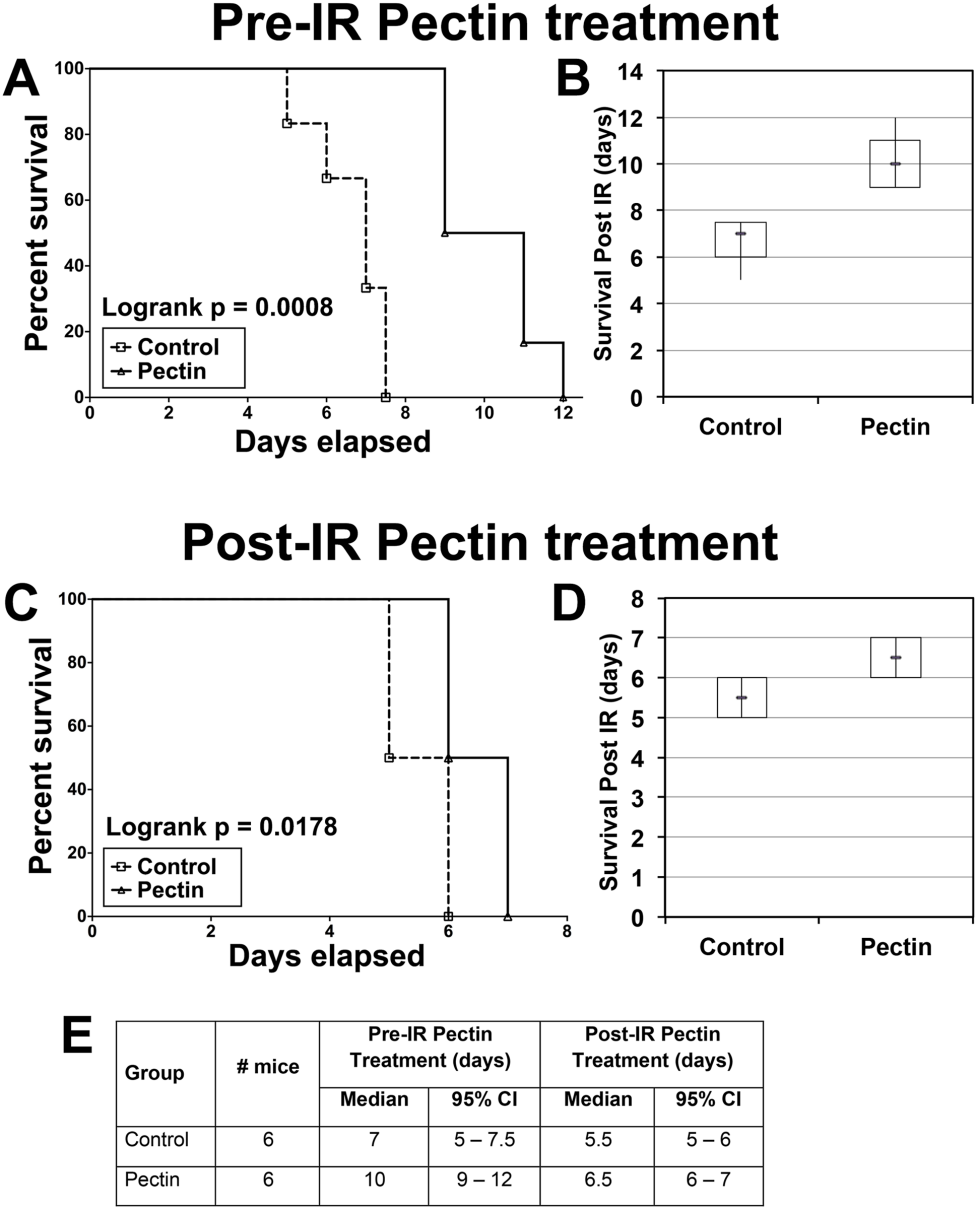
Pectin delays radiation-induced death in mice. (A and B) Pre-IR pectin treatment: C57BL/6 mice were pretreated with either control or pectin diet for one week prior to 14 Gy IR injury (n = 6 in each group). Overall survival of the animals is represented in the graph and the overall survival rate is significantly higher among pectin pretreated mice compared to controls (A). Side-by-side box plot of overall survival data for pectin pretreated mice and control mice (B). (C and D) Post-IR pectin treatment: C57BL/6 mice were subjected to 14 Gy IR injury and fed with control or pectin diet (n = 6 in each group). Overall survival of the animals is represented in the graph and the overall survival rate is significantly higher among pectin post-treated mice compared to controls (C). Side-by-side box plot of overall survival data for pectin post-treated mice and control mice (D). (E) Table represents the Median and the 95% CI values in the pre-IR pectin treatment and post-IR pectin treatments. Control mice in the pre-IR pectin treatments had a median survival of 7 d with a 5–7.5 d 95% CI; Pectin treated mice had a median survival of 10 d with a 9–12 d 95% CI. In the post-IR pectin groups, control mice had a median survival of 5.5 d with a 5–6 d 95% CI; pectin treated animals had a median survival of 6.5 d with a 6–7 d 95% CI. Both the groups had n = 6 mice.

C57BL/6 mice (n = 6) on control diet died at an average of 6.7 days (with a median value of 7 days and 95% CI = 5–7.5 days) after treatment, whereas mice (n = 6) pretreated with pectin diet on average lived beyond 10 days (with a median value of 10 days and 95% CI = 9–12 days) post-14 Gy IR (Fig 3A, 3B and 3E). We observed a significant difference (logrank p = 0.0008) in the survival curve of control and pectin-treated animals (Fig 3A).

#### Post-IR pectin treatment

To demonstrate the effect of post-IR pectin treatment, animals were initially treated with 14 Gy TBI and fed with control (n = 6) or pectin (n = 6) diet (S1 Fig). Mice on control diet died at an average of 5.5 days (with a median value of 5.5 days and 95% CI = 5–6 days) after radiation, whereas mice treated with pectin diet died at an average of 6.5 days (with a median value of 6.5 days and 95% CI = 6–7 days) post-14 Gy IR (Fig 3C, 3D and 3E). This difference in survival rates was statistically significant (logrank p = 0.0178) (Fig 3C).

### Pectin protects ISCs following radiation injury

**Fig 4:**
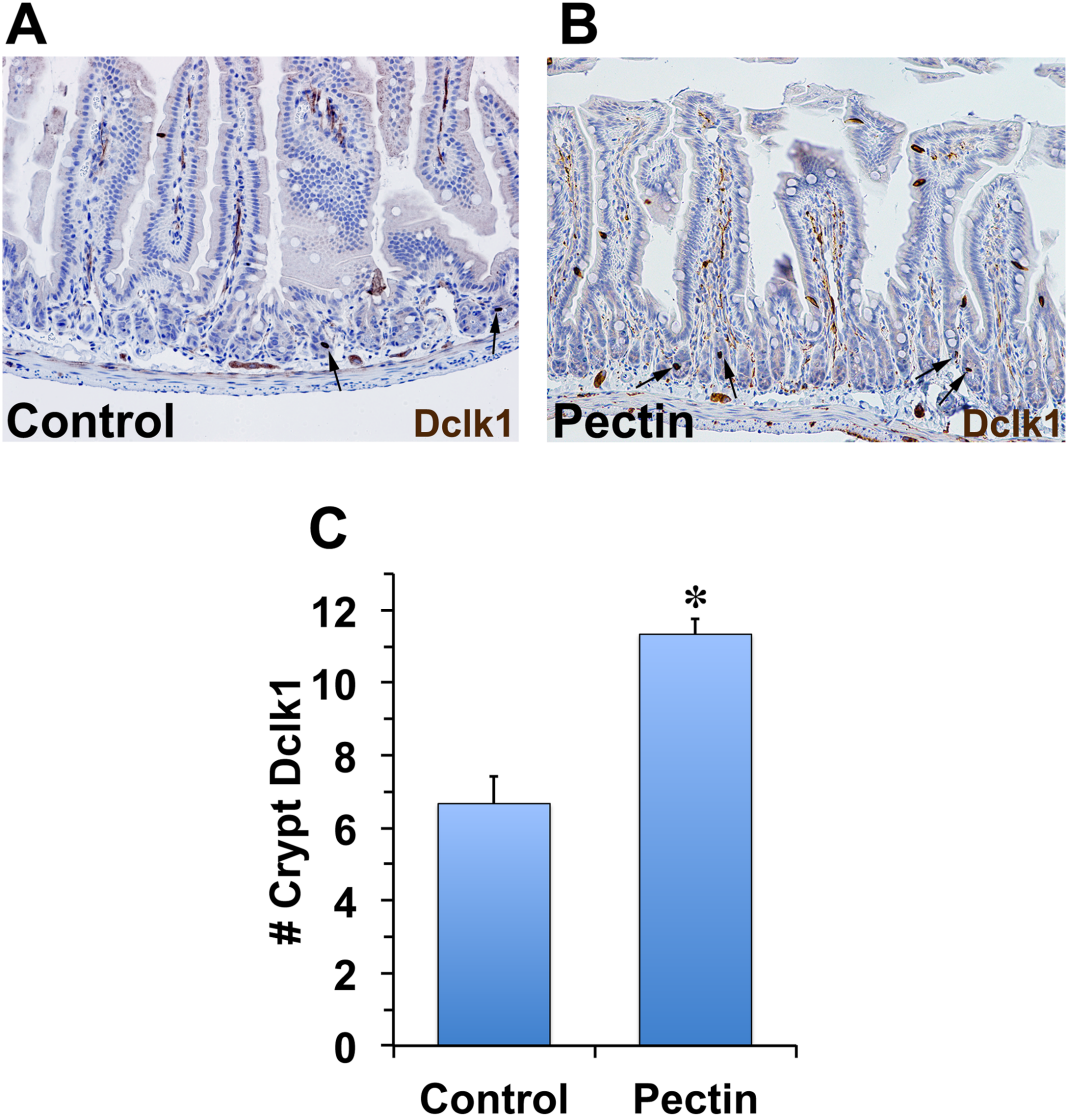
Pectin treatment increased the number of surviving Dclk1 positive cells 24h post-IR. C57BL/6 mice in the control group (A) and the pectin-treated group (B) were subjected to 14 Gy TBI. 24h post-IR, the small intestines were isolated, fixed, processed, and stained with anti-Dclk1 antibody (n = 3 in each group). Dclk1 positive cells in cross section are indicated by arrows in panels A and B. The average number of Dclk1 positive cells per cross section was counted for total of 30 cross sections and presented in panel C. The values in the bar graph are given as average ± SEM and * denotes statistically-significant differences (*p<0.01) compared to control.

In order to demonstrate whether ISCs are protected and are responsible for the increased crypt and overall survival of mice pretreated with pectin, we performed mRNA and protein analysis of genes that are known to play an important role in stem cells. Results from our previous studies demonstrated that the number of Dclk1+ stem cells at 24h post-IR is an indicator of surviving crypts at 84h post-IR [22]. We used Dclk1 as the ISC marker. Previously, we demonstrated that Dclk1+ cells undergo apoptosis at 24h post-high dose IR. Control and pectin-treated animals were euthanized at 24h post-IR. Intestines were then fixed and subjected to Dclk1 immunostaining. In the IR-treated group, we observed an average of 6.6 Dclk1+ stem cells per cross section (counted 10 cross sections per mouse and 3 mice in each group) (Fig 4A and 4C). The pectin pre-treatment group had a nearly two-fold increase in crypt Dclk1+ stem cells (11.3 cells per cross section) (n = 3) (Fig 4B and 4C). These data suggest that pretreatment of mice with pectin has protective effects against radiation-induced injury to ISCs.

### Increased expression of intestinal stem cell genes following pectin pre-treatment

**Fig 5:**
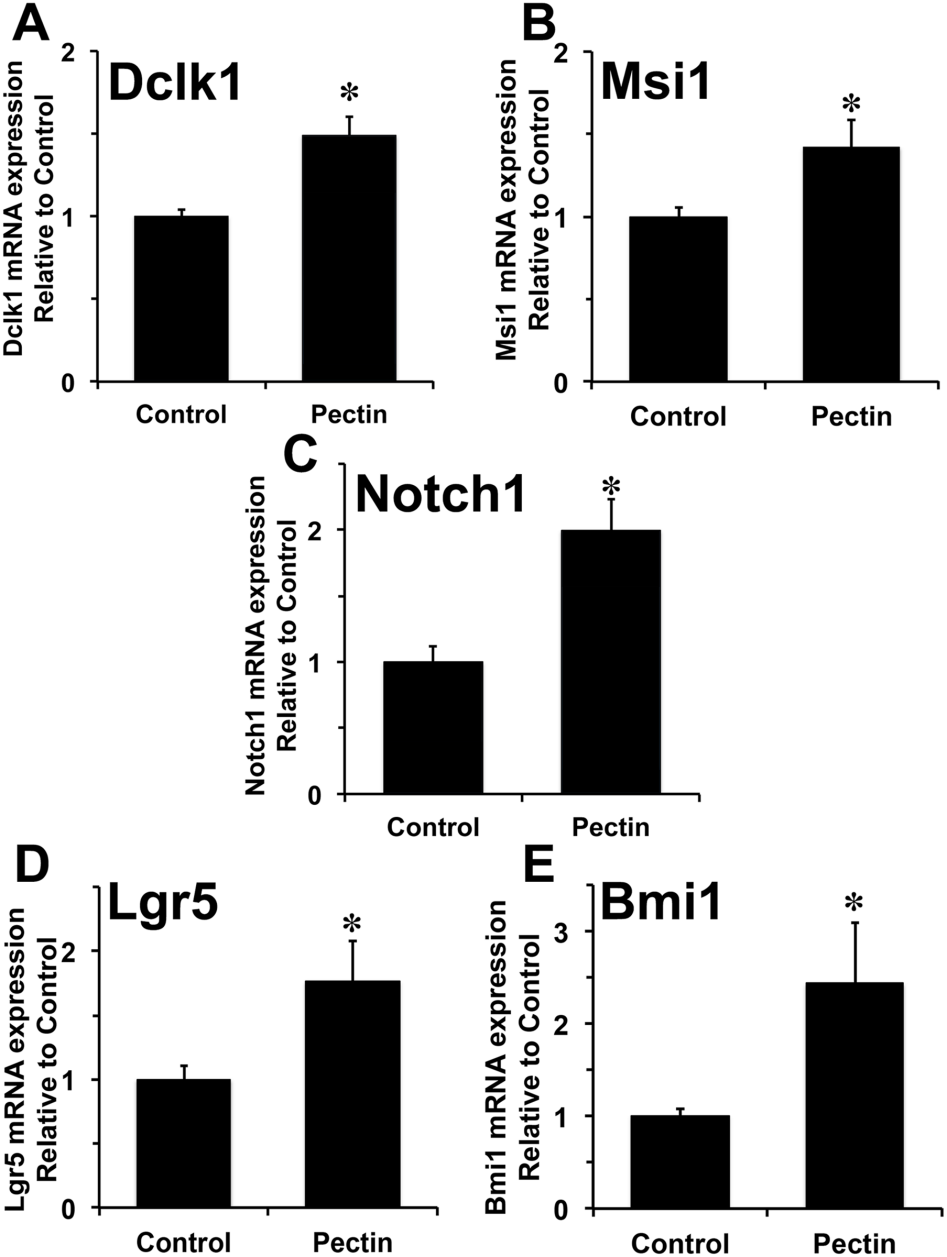
Pectin treatment increased Dclk1, Notch1, Msi1, Lgr5, and Bmi1 expression 24h post-IR. Total RNAs isolated from the small intestine after each treatment were subjected to quantitative RT-PCR. Dclk1 (A), Notch1 (B), Msi1 (C), Lgr5 (D), and Bmi1 (E) mRNA expression following control and pectin treatment are depicted. The values in the bar graph are given as average ± SEM and * denotes statistically-significant differences (*p<0.05) compared to control (n = 3 in each group).

Total RNA isolated from the intestines of 24h post-IR samples (n = 3) was subjected to RT-PCR for stem cell related genes such as Dclk1, Msi1, Lgr5, Bmi1, and Notch1. We observed significant upregulation of Dclk1 (1.5-fold) in mice pretreated with pectin compared to control mice (Fig 5A). Similarly, we found significant upregulation of Msi1, an intestinal stem/progenitor cell marker, in the pectin group compared to control (Fig 5B). We also observed a significant upregulation of Notch1 (two fold) in the pectin group (Fig 5C). Additionally, we also observed increased expression of putative ISC markers Lgr5 (1.76-fold) (Fig 5D) and Bmi1 (2.43-fold) (Fig 5E) in the pectin group. These data indicate that pectin treatment resulted in an increase in stem cell-related genes, probably in the stem cells resulting in increased crypt survival and overall survival.

## Discussion

The two most radiosensitive human tissues that are also essential for sustaining life are the GI epithelium and the hematopoietic progenitor cells in the bone marrow [23, 24]. Death due to effects of IR on GI can be measured by the survival of the animal up to ten days post high-dose IR; the greater the IR dose, the shorter the survival period. If animals survive more than ten days after exposure, death is due to the loss of hematopoietic stem cells [23, 24]. The GI response to acute radiation injury is the most extensively characterized model system for studying injury-repair in the rodent. The continuous and rapid turnover of the GI tract explains its unique sensitivity to high doses of IR. Following high dose IR (>8 Gy), GI stem/progenitor epithelial cells are unable to multiply at a rate sufficient to replace dying cells. This failure results in malabsorption syndromes, electrolyte imbalances, fluid loss, and bacterial infections [2, 3]. Agents that can prevent or attenuate epithelial stem/progenitor cell loss, as well as restore epithelial integrity, would be ideal candidates for radioprotective drug development.

Radiation therapy for various cancers has been one of the most effective treatment options. Patients undergoing radiotherapy suffer adverse effects as stated above. Prevention of damage during treatment can be achieved by administering radioprotectors prior to radiation. Potential use of chemicals to protect against the harmful effects of radiation was investigated after World War II. These chemicals included amino-acid cysteine and thiol compounds [24]. One such compound Amifostine (an organic thiophosphate), is a pretreatment for some patients undergoing radiation therapy. Amifostine has serious side effects however, including hypotension, which seriously limits its use. Androstenediol has recently been proposed as a prophylactic drug; however, the detailed toxicity evaluation for this drug is still in progress [25]. The high toxicity of these compounds has created a demand for alternative nontoxic radioprotectors.

In this study, mice pre-treated with dietary citrus pectin (in animal feed and drinking water) were subjected to high-dose IR (14 Gy). Pectin increased intestinal crypt survival and increased expression of Dclk1, Msi1, and Notch1 (which play important roles in putative stem cells and differentiation). Recently, we have demonstrated that mice with intestinal and colonic specific knockout of Dclk1 had impaired regenerative response following high-dose TBI died early compared to their wild type littermates [14]. Here, we observed increased numbers of Dclk1+ cells and increased expression of genes (known to protect or play crucial role in ISCs) in the intestine of the mice treated with pectin. We believe these protecting actions of pectin resulted in increased crypt survival at 84h, and were responsible for maintaining the epithelial integrity in the animal’s increased overall survival. This also was supported by our previous publication, where that number of crypt Dclk1+ cells at 24h post-IR directly correlated with the crypt survival at 84h [22]. Additionally, we have demonstrated that inhibition of Notch signaling resulted in nearly 50% reduction in the number of surviving crypt Dclk1+ cells at 24h and similar reduction in number of surviving crypts at 84h post-IR. These data indicated that Notch signaling decreases ISC survival following IR treatment, suggesting an important role of Notch in ISC-mediated crypt regeneration [22]. In this study, Notch mRNA was upregulated in the mouse intestines after pectin treatment, suggesting that pectin might have protected the ISCs and pathways regulating ISC survival.

Musashi-1 (Msi1), an RNA binding protein, is a putative intestinal stem/progenitor cell marker and it is expressed in the stem cell zone of the mouse intestine [26–28]. Furthermore, knockdown of Msi1 lead to mitotic catastrophe in tumor cells and resulted in inhibition of Notch1, indicating that Msi1 regulates Notch signaling [29]. Previously, it has been demonstrated that p21^-/-^ mice had increased Msi1 in regenerative crypts (84h post-IR) compared to its littermates [10]. Additionally, p21^-/-^ mice had increased crypt survival (3-fold) compared to its littermates [10]. These data taken together suggest that Msi1 may play an important role in ISC-mediated regenerative crypts following IR injury. Due to these reasons, we wanted to explore the expression levels of Msi1 in this study. We observed increased expression of Msi1 in the intestines of mice treated with pectin compared to control diet, supporting the conclusion that pectin protected ISCs following lethal TBI. Furthermore, other putative ISC markers Lgr5 (proliferative ISC) and Bmi1 (quiescent ISC) were also upregulated in the intestines of mice treated with pectin compared to control, confirming that pectin indeed protected ISCs following lethal TBI.

Previously published studies suggests that purified cellulose, 6% pectin or hemicellulose fiber diets increased intestinal and colonic crypt height, and mitotic index [18, 30–32]. These data taken together lead to our current hypothesis that 6% pectin would protect the intestinal epithelial and stem cells against IR injury. Indeed, pectin treatment exerted radioprotective properties by protecting the ISCs and ISCs-derived regenerative crypts following high dose IR. This major outcome of the present study enabled us to directly determine the effects of radioprotective and/or radiation mitigators on stem cell fate. Furthermore in our studies, we observed significant anti-inflammatory property of pectin following bacterial infection [18, 30–32]. Based on that and the outcome of this study, we speculate that pectin protects the ISCs and the intestinal epithelial cells against inflammation, hyperplasia of lamina propria and probably increases intestinal tight junction protein like ZO1, ZO2, Claudin1, and Claudin 7 (which are downstream Dclk1)[14] to preserve the barrier. In conclusion, pectin was shown to be an important candidate as a radioprotectant. Treatment with pectin had beneficial effects on gut protection and overall survival following severe TBI. Additionally (given the anti-cancer activity of pectin), these data taken together support a role for dietary pectin as an agent that can be administered to patients receiving radiation therapy to protect against radiation-induces mucositis. As a future direction, we are in the process of evaluating the role of pectin on the intestinal junction proteins, maintenance of barrier, and also on prevention of DNA damage following IR injury. Additionally, we are also extending these studies to include varying doses of pectin and IR (whole-body and abdominal-focused IR injury) to achieve an optimal protective dose of pectin. Finally, we are also evaluating the role of various synthetic compounds (radiation mitigators) on ISC and crypt survival/regeneration using this established animal model.

## Supporting Information

S1 Checklist The ARRIVE Guidelines Checklist. (PDF)

S1 Fig Schematic presentation of experimental design (post-IR pectin treatment). C57BL/6 mice in the control group (A) or the pectin treated group (B) were fed with control or pectin diet and concurrently subjected to 14 Gy TBI (0 h). For the microcolony assay, one set of mice were administered BrdUrd at 82 h post-IR and killed at 84h. The other set of mice were subjected to overall animal survival studies up to 288h post-IR—times of death were noted.
(TIF)

## References

[1] Avritscher EB, Cooksley CD, Elting LS. Scope and epidemiology of cancer therapy-induced oral and gastrointestinal mucositis. Seminars in oncology nursing. 2004;20(1):3–10. . 15038511 10.1053/j.soncn.2003.10.002

[2] Yeoh E, Horowitz M, Russo A, Muecke T, Robb T, Maddox A, et al Effect of pelvic irradiation on gastrointestinal function: a prospective longitudinal study. The American journal of medicine. 1993;95(4):397–406. . 8213872 10.1016/0002-9343(93)90309-d

[3] Yeoh EK, Russo A, Botten R, Fraser R, Roos D, Penniment M, et al Acute effects of therapeutic irradiation for prostatic carcinoma on anorectal function. Gut. 1998;43(1):123–7. 9771416 10.1136/gut.43.1.123 PMC1727195

[4] Stringer AM, Gibson RJ, Bowen JM, Logan RM, Yeoh AS, Keefe DM. Chemotherapy-induced mucositis: the role of gastrointestinal microflora and mucins in the luminal environment. The journal of supportive oncology. 2007;5(6):259–67. . 17624050

[5] Keefe DM, Gibson RJ, Hauer-Jensen M. Gastrointestinal mucositis. Seminars in oncology nursing. 2004;20(1):38–47. . 15038516 10.1053/j.soncn.2003.10.007

[6] Koukourakis MI. Radiation damage and radioprotectants: new concepts in the era of molecular medicine. The British journal of radiology. 2012;85(1012):313–30. 10.1259/bjr/16386034 22294702 PMC3486665

[7] Gordon JI, Hermiston ML. Differentiation and self-renewal in the mouse gastrointestinal epithelium. Current opinion in cell biology. 1994;6(6):795–803. . 7880525 10.1016/0955-0674(94)90047-7

[8] Potten CS, Booth C, Pritchard DM. The intestinal epithelial stem cell: the mucosal governor. International journal of experimental pathology. 1997;78(4):219–43. 9505935 10.1046/j.1365-2613.1997.280362.x PMC2694540

[9] May R, Riehl TE, Hunt C, Sureban SM, Anant S, Houchen CW. Identification of a novel putative gastrointestinal stem cell and adenoma stem cell marker, doublecortin and CaM kinase-like-1, following radiation injury and in adenomatous polyposis coli/multiple intestinal neoplasia mice. Stem cells. 2008;26(3):630–7. 10.1634/stemcells.2007-0621 . 18055444

[10] George RJ, Sturmoski MA, May R, Sureban SM, Dieckgraefe BK, Anant S, et al Loss of p21Waf1/Cip1/Sdi1 enhances intestinal stem cell survival following radiation injury. American journal of physiology Gastrointestinal and liver physiology. 2009;296(2):G245–54. 10.1152/ajpgi.00021.2008 19056768 PMC2643902

[11] Gerbe F, van Es JH, Makrini L, Brulin B, Mellitzer G, Robine S, et al Distinct ATOH1 and Neurog3 requirements define tuft cells as a new secretory cell type in the intestinal epithelium. The Journal of cell biology. 2011;192(5):767–80. Epub 2011/03/09. 10.1083/jcb.201010127 21383077 PMC3051826

[12] Short B. A fifth amendment to the intestine's constitution. The Journal of cell biology. 2011;192(5):706-. 10.1083/jcb.1925iti2 PubMed Central PMCID: PMCPMC3051811.

[13] Gerbe F, Legraverend C, Jay P. The intestinal epithelium tuft cells: specification and function. Cellular and molecular life sciences: CMLS. 2012;69(17):2907–17. Epub 2012/04/25. 10.1007/s00018-012-0984-7 22527717 PMC3417095

[14] May R, Qu D, Weygant N, Chandrakesan P, Ali N, Lightfoot SA, et al Brief report: Dclk1 deletion in tuft cells results in impaired epithelial repair after radiation injury. Stem cells. 2014;32(3):822–7. 10.1002/stem.1566 . 24123696 PMC4603545

[15] Citrin D, Cotrim AP, Hyodo F, Baum BJ, Krishna MC, Mitchell JB. Radioprotectors and mitigators of radiation-induced normal tissue injury. The oncologist. 2010;15(4):360–71. 10.1634/theoncologist.2009-S104 20413641 PMC3076305

[16] Voragen AGJ, Coenen GJ, Verhoef RP, Schols HA. Pectin, a versatile polysaccharide present in plant cell walls. Struct Chem. 2009;20(2):263–75. 10.1007/S11224-009-9442-Z WOS:000264880400014.

[17] Nangia-Makker P, Hogan V, Honjo Y, Baccarini S, Tait L, Bresalier R, et al Inhibition of human cancer cell growth and metastasis in nude mice by oral intake of modified citrus pectin. Journal of the National Cancer Institute. 2002;94(24):1854–62. . 12488479 10.1093/jnci/94.24.1854

[18] Umar S, Morris AP, Kourouma F, Sellin JH. Dietary pectin and calcium inhibit colonic proliferation in vivo by differing mechanisms. Cell proliferation. 2003;36(6):361–75. . 14710853 10.1046/j.1365-2184.2003.00291.x PMC6496283

[19] Sureban SM, May R, Ramalingam S, Subramaniam D, Natarajan G, Anant S, et al Selective blockade of DCAMKL-1 results in tumor growth arrest by a Let-7a MicroRNA-dependent mechanism. Gastroenterology. 2009;137(2):649–59, 59 e1–2. 10.1053/j.gastro.2009.05.004 19445940 PMC2775069

[20] Sureban SM, May R, Qu D, Weygant N, Chandrakesan P, Ali N, et al DCLK1 regulates pluripotency and angiogenic factors via microRNA-dependent mechanisms in pancreatic cancer. PloS one. 2013;8(9):e73940 10.1371/journal.pone.0073940 24040120 PMC3767662

[21] Sureban SM, May R, Weygant N, Qu D, Chandrakesan P, Bannerman-Menson E, et al XMD8-92 inhibits pancreatic tumor xenograft growth via a DCLK1-dependent mechanism. Cancer letters. 2014;351(1):151–61. 10.1016/j.canlet.2014.05.011 . 24880079

[22] Qu D, May R, Sureban SM, Weygant N, Chandrakesan P, Ali N, et al Inhibition of Notch signaling reduces the number of surviving Dclk1+ reserve crypt epithelial stem cells following radiation injury. American journal of physiology Gastrointestinal and liver physiology. 2014;306(5):G404–11. 10.1152/ajpgi.00088.2013 24368703 PMC3949020

[23] Jagetia GC, Baliga MS. Evaluation of the radioprotective effect of the leaf extract of Syzygium cumini (Jamun) in mice exposed to a lethal dose of gamma-irradiation. Die Nahrung. 2003;47(3):181–5. 10.1002/food.200390042 . 12866620

[24] G CJ. Radioprotective Potential of Plants and Herbs against the Effects of Ionizing Radiation. Journal of clinical biochemistry and nutrition. 2007;40(2):74–81. 10.3164/jcbn.40.74 18188408 PMC2127223

[25] Xiao M, Inal CE, Parekh VI, Chang CM, Whitnall MH. 5-Androstenediol promotes survival of gamma-irradiated human hematopoietic progenitors through induction of nuclear factor-kappaB activation and granulocyte colony-stimulating factor expression. Molecular pharmacology. 2007;72(2):370–9. 10.1124/mol.107.035394 . 17473057

[26] Yu T, Chen QK, Gong Y, Xia ZS, Royal CR, Huang KH. Higher expression patterns of the intestinal stem cell markers Musashi-1 and hairy and enhancer of split 1 and their correspondence with proliferation patterns in the mouse jejunum. Medical science monitor: international medical journal of experimental and clinical research. 2010;16(2):BR68–74. . 20110912

[27] Potten CS, Booth C, Tudor GL, Booth D, Brady G, Hurley P, et al Identification of a putative intestinal stem cell and early lineage marker; musashi-1. Differentiation; research in biological diversity. 2003;71(1):28–41. . 12558601 10.1046/j.1432-0436.2003.700603.x

[28] Dekaney CM, Rodriguez JM, Graul MC, Henning SJ. Isolation and characterization of a putative intestinal stem cell fraction from mouse jejunum. Gastroenterology. 2005;129(5):1567–80. 10.1053/j.gastro.2005.08.011 . 16285956

[29] Sureban SM, May R, George RJ, Dieckgraefe BK, McLeod HL, Ramalingam S, et al Knockdown of RNA binding protein musashi-1 leads to tumor regression in vivo. Gastroenterology. 2008;134(5):1448–58. 10.1053/j.gastro.2008.02.057 . 18471519

[30] Dirks P, Freeman HJ. Effects of differing purified cellulose, pectin and hemicellulose fiber diets on mucosal morphology in the rat small and large intestine. Clinical and investigative medicine Medecine clinique et experimentale. 1987;10(1):32–8. . 3028682

[31] Chandrakesan P, Ahmed I, Anwar T, Wang Y, Sarkar S, Singh P, et al Novel changes in NF-{kappa}B activity during progression and regression phases of hyperplasia: role of MEK, ERK, and p38. The Journal of biological chemistry. 2010;285(43):33485–98. 10.1074/jbc.M110.129353 20710027 PMC2963366

[32] Chandrakesan P, Ahmed I, Chinthalapally A, Singh P, Awasthi S, Anant S, et al Distinct compartmentalization of NF-kappaB activity in crypt and crypt-denuded lamina propria precedes and accompanies hyperplasia and/or colitis following bacterial infection. Infection and immunity. 2012;80(2):753–67. 10.1128/IAI.06101-11 22144489 PMC3264290

