# Supplementary figures and images for: Dietary Pectin Increases Intestinal Crypt Stem Cell Survival following Radiation Injury
Source: PLoS One. 2015 Aug 13;10(8):e0135561. doi: 10.1371/journal.pone.0135561 (PMC4536042; doi:10.1371/journal.pone.0135561)

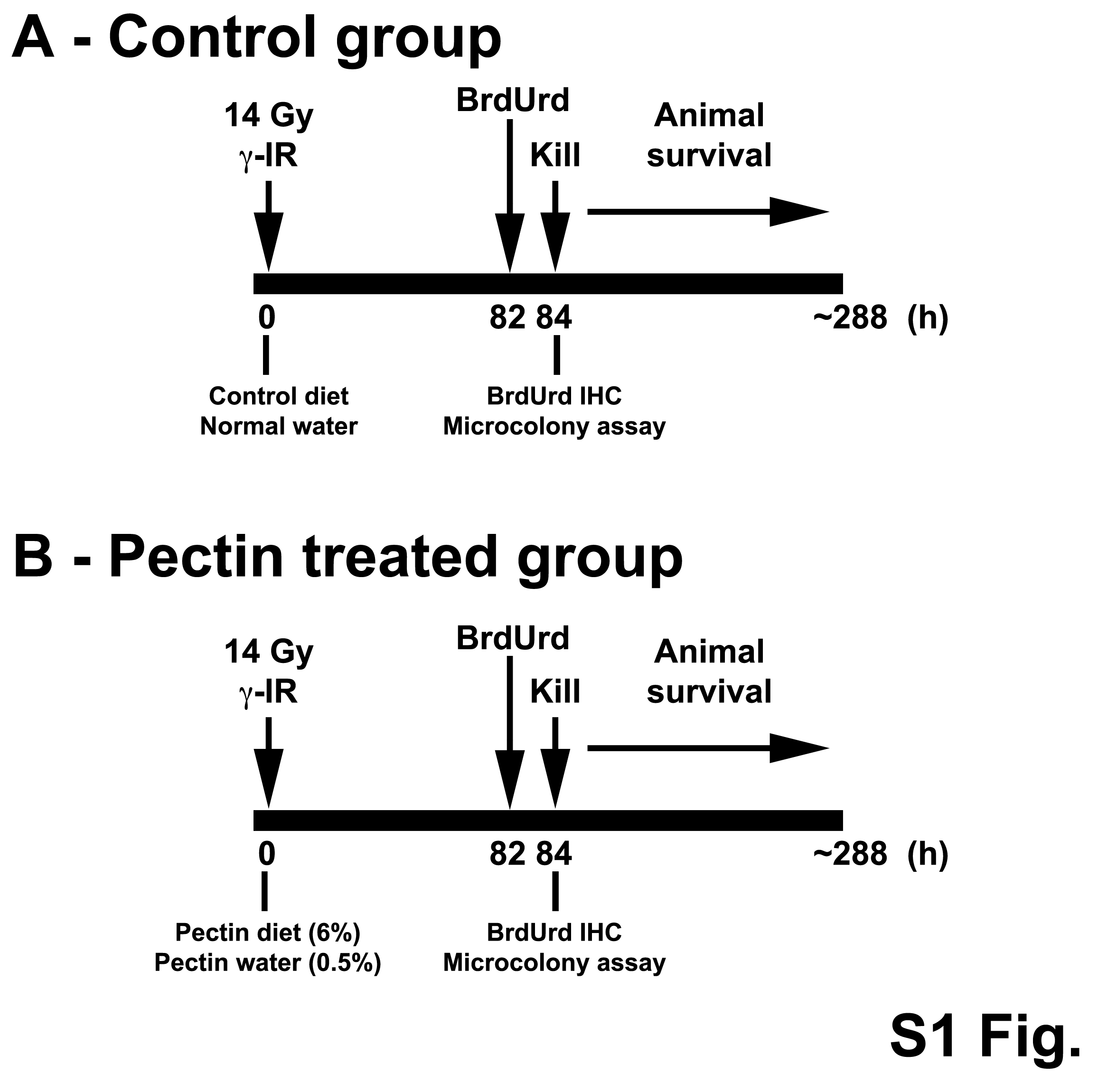

Supplement: S1 Fig — C57BL/6 mice in the control group (A) or the pectin treated group (B) were fed with control or pectin diet and concurrently subjected to 14 Gy TBI (0 h). For the microcolony assay, one set of mice were administered BrdUrd at 82 h post-IR and killed at 84h. The other set of mice were subjected to overall animal survival studies up to 288h post-IR—times of death were noted. (TIF) [file pone.0135561.s002.tif]
